# Circumferential 2D-strain imaging for the prediction of long term response to cardiac resynchronization therapy

**DOI:** 10.1186/1476-7120-6-28

**Published:** 2008-06-06

**Authors:** Fabian Knebel, Sebastian Schattke, Hansjürgen Bondke, Stephan Eddicks, Andrea Grohmann, Gert Baumann, Adrian C Borges

**Affiliations:** 1 Universitätsmedizin Berlin, Medical Clinic for Cardiology and Angiology, Charité Campus Mitte, Charitéplatz 1, 10098 Berlin, Germany

## Abstract

**Background:**

Cardiac Resynchronization Therapy (CRT) leads to hemodynamic and clinical improvement in heart failure patients. The established methods to evaluate myocardial asynchrony analyze longitudinal and radial myocardial function. This study evaluates the new method of circumferential 2D-strain imaging in the prediction of the long-term response to CRT.

**Methods and results:**

38 heart failure patients (NYHA II-III, QRS > 120 ms, LVEF < 0.35) received CRT and echocardiographic evaluation with a mean follow-up of 9.4 months. 18 (47.4%) of the patients were hemodynamic responders to long-term CRT. In the responder group, the maximum delay in the circumferential 2D-strain in the basal segments decreased (246 ± 94 to 123 ± 92 ms, p < 0.001). In the non-responder group there was no significant change (pre CRT: 195 ± 86, post CRT 135 ± 136 ms, p = 0.84). This was paralleled by a reduction of the maximum delay in the radial and longitudinal 2D strain in the basal segments. In ROC analysis, the baseline delay of circumferential 2D strain (AUC 0.66 (± 0.14)) does not predict a long-term response to CRT (p = 0.37).

**Conclusion:**

There is a significant decrease in the circumferential 2D-strain derived delays after CRT, indicating that resynchronization induces improvement in all three dimensions of myocardial contraction. However, the resulting predictive values of 2D strain delays are not superior to longitudinal and radial 2D-strain or TDI delays.

## Background

Cardiac resynchronisation therapy (CRT) is an effective therapy for advanced chronic heart failure. Randomized, controlled trials indicate that CRT improves hemodynamics, reverse remodelling, quality of life, hospitalisation and mortality [1-7]. However, in the large CRT studies, the responder rates were low (43–63%) [6,8,9]. In the CARE-HF (2005): about 50% of the patients responded clinically. The COMPANION study did not publish the responder rates (2004) [1].

In this context, it is essential to select patients for CRT carefully and to define predictors. Several studies have focused on prospectively predicting successful CRT by echocardiography. However, only parameters of longitudinal and radial myocardial function were used [8,10].

There are conflicting data about the best predictor for hemodynamic and clinical improvement. Several echocardiographic (including 3D echocardiography) and clinical predictors were assessed recently [10-14].

Myocardial contraction is a complex three-dimensional motion. Fibre architecture includes longitudinal, radial and circumferential oriented fibres. The majority of fibres have a longitudinal orientation. However, radial and circumferential fibres contribute to myocardial systolic function [15,16].

Circumferential myocardial function can be analysed by 2D ("speckle tracking") derived echocardiography. Unlike Tissue Doppler derived velocity and strain, 2D-derived analysis [17] is angle-independent and allows the measurement of circumferential strain [18,19].

The aim of this study is to examine the improvement of circumferential myocardial contractility after CRT is analysed.

## Patients and methods

In this monocentric study, we included 38 heart failure patients (NYHA II-IV) with a LVEF < 0.35 and a QRS width > 120 ms. All patients received a CRT-device with automatic defibrillator (ICD) function and were fully documented by 2D and Tissue Doppler echocardiography. Follow-up was at least 6 months. The heart failure medication was unchanged three months prior to implantation of the CRT-ICD to obtain unbiased data regarding cardiac improvement after CRT.

Successful resynchronization therapy was defined as a relative reduction of the left-ventricular end-systolic volume (LV-ESV) of more than 15% [20,21] and a relative increase of the LVEF of more than 25% compared to baseline [22]. The latter has been evaluated to a follow-up interval of 6 months.

Echocardiography was performed by Vivid 5 and Vivid 7 (GE Vingmed, Horton, Norway). The images were stored digitally and analyzed off-line by EchoPac PC Dimension (GE Vingmed, Horton Norway). For TDI and 2D echocardiography analysis, three beats were stored.

The echocardiographic study design was described previously [11]. The LVEF and left-ventricular volumes were calculated according to Simpson's rule [23].

Circumferential 2D strain was measured in the parasternal short axis at the level of the papillary muscles. The endocardial border of the end-systole was traced manually. The following six segments were analysed: antero-septal, anterior, lateral, posterior, inferior, septal. The maximal delays in opposite segments (anterior-septal/posterior, anterior/inferior, septal/lateral) were then calculated. The maximum delay was then used for further analyses.

The maximal longitudinal delays were obtained from apical and the maximal radial delays were measured in the parasternal short axis views.

Written consent was obtained from each patient, and the ethics committee of the Charité University Hospital approved the protocol.

Statistics were calculated by SPSS (version 12.0, Chicago, Ill, USA). Results are expressed as mean (± standard deviation). Comparisons of parametric variables between the responders and the non-responders were calculated by paired Student's t-test. The comparison of echocardiographic parameters between groups was calculated by unpaired t-test. Dichotomized data were analyzed by the Chi^2^-test. The level of significance was p = 0.05.

## Results

### Patients

Eighteen (47.4%) of the 38 patients were hemodynamic responders to long-term CRT. The baseline characteristics in the responder and non-responder group did not differ significantly. Baseline characteristics see Table 1.

**Table 1:** Patient characteristics at baseline

	**All (n = 38)**	**Responders (n = 18)**	**Non-Responders (n = 20)**	**Difference responders vs. non-responders [p]**
**Age [years]**	63.6 (± 10.8)	65.5 (± 8.0)	64.9 (± 11.6)	0.85
**QRS width [ms]**	165.1 (± 18.4)	160.3 (± 10.6)	170.8 (± 23.9)	0.15
**Echo follow-up [months]**	9.4 (± 3.0)	9.5 (± 3.3)	9.7 (± 2.5)	0.82
**Sex [% male]**	68.4	65.0	72.2	0.63
**Etiology of heart failure [% ischemic]**	21.1	20.0	20.0	0.71
**LVEF at baseline**	0.25 (± 8.0)	0.23 (± 8.7)	0.26 (± 6.8)	0.24
**LV-ESV [ml]**	199,04 (± 89.4)	185.3 (± 80.6)	213.6 (± 98.0)	0.35

### Hemodynamics

The hemodynamics of the responder and non-responder group: see Table 2. After CRT, there is a significant reverse remodelling in the responder group. The placement of the left ventricular lead was in posterolateral position in 52.6% of the patients. The lead placement was not different in the responder and the non-responder group (p = 0.34).

**Table 2:** Hemodynamics TDI-derived velocity and strain and 2D-strain echocardiography at baseline and after follow-up.

	**Responder**			**Non-Responder**		
	**BASELINE**	**FOLLOW-UP**	**p**	**BASELINE**	**FOLLOW-UP**	**P**

**LVEF**	0.23	0.36	< 0.01	0.26	0.24	0.12
**LVESV [ml]**	185.3	136.6	0.01	213.6	228.3	0.34
**s-wave [cm/s]**	3.26 (± 0.50)	3.64 (± 1.20)	0.39	2.62 (± 0.32)	3.06 (± 3.06)	0.30
**TDI strain [%]**	18.62 (± 5.61)	15.76 (± 3.79)	0.22	18.40 (± 4.01)	20.99 (± 6.11)	0.34
**Max. delay radial [ms]**	167.6 (± 104.4)	98.1 (± 43.6)	**0.04**	178.7 (± 92.2)	144.5 (± 122.5)	0.75
**Max. delay longitudinal [ms]**	167.5 (± 90.5)	111.7 (± 80.6)	**0.02**	217 (± 125)	152 (± 132)	0.15
**Max. delay cicumferential [ms]**	246.1 (± 94.4)	123.3 (± 92.52)	**0.00023**	195.0 (± 85.6)	135.18 (± 136.09)	0.84
**Mean circumferential 2D strain [%]**	-6.7 (± 2.9)	-8.0 (± 2.4)	0.13	-6.38 (± 1.7)	-5.9 (± 3.2)	0.46
**Mean longitudinal 2D strain [%]**	5.6 (± 2.6)	6.4 (± 3.6)	0.49	5.2 (± 1.7)	5.0 (± 1.8)	0.83
**Mean radial 2D strain [%]**	12.1 (± 6.8)	12.8 (± 7.5)	0.49	9.8 (± 5.1)	8.9 (± 5.6)	0.87

### 2D strain echocardiography

**Figure 1:**
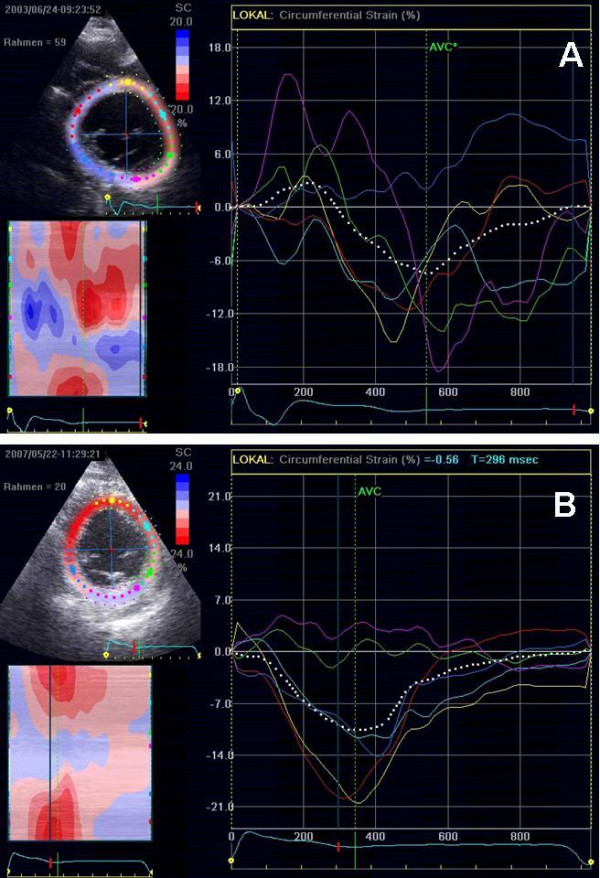
**Circumferential 2D strain before (**A**) and after (**B**) 4 years of CRT in a responder.** Parasternal short axis view at the level of the papillary muscles. Before CRT, there is asynchronous circumferential contraction with postsystolic shortening and passive movement of the inferior and posterior segments, which are scar tissue. After 4 years of CRT, there is increased synchronous contraction, a reduction of postsystolic shortening; the scarred segments (inferior and posterior) show no circumferential contraction.

**Figure 2:**
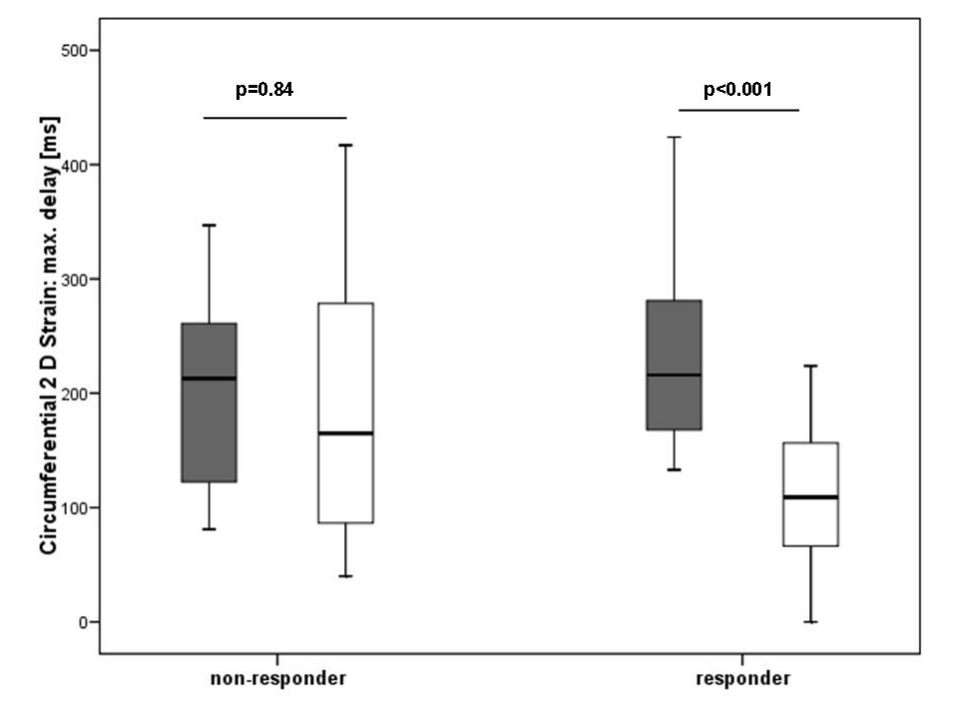
Boxplot analysis of the maximum delays in circumferential 2D strain at baseline (grey boxes) and follow up (white boxes) in the non-responder and responder to CRT.

The maximum delay in the circumferential, longitudinal and radial 2D-strain decreased significantly in the responder group but not in the non-responder group (Table 3, Figure 2). Figure 1 illustrates a responder in the long-term follow-up with improvement of circumferential synchrony. Two videoclips showing circumferential 2D strain in the parasternal short axis of a patient with DCM (**See additional file 1**) and a healthy control (**See additional file 2**) are attached.

**Table 3:** Areas under the curve (AUC) in the receiver-operating characteristics (ROC) analysis for the prediction of benefit from CRT

	**AUC (± SD)**
**TDI-velocity**	0.696(± 0.116)
**2D circumferential strain**	0.661 (± 0.144)
**TDI-strain**	0.546 (± 0.126
**2D radial strain**	0.432 (± 0.119)
**2D longitudinal strain**	0.368 (± 0.121)
**QRS width**	0.341 (± 0.092)

### Tissue Doppler echocardiography

The maximum delay in the peak systolic velocities in the basal segments decreased in the responder group but not in the non-responder group. The maximum delay in the peak systolic strain in the basal segments did not change significantly in the responder group or in the non-responder group. The s-wave velocity in the basal septum and the TDI-based strain did not change significantly in the two patient groups (Table 2).

### ROC analysis

**Figure 3:**
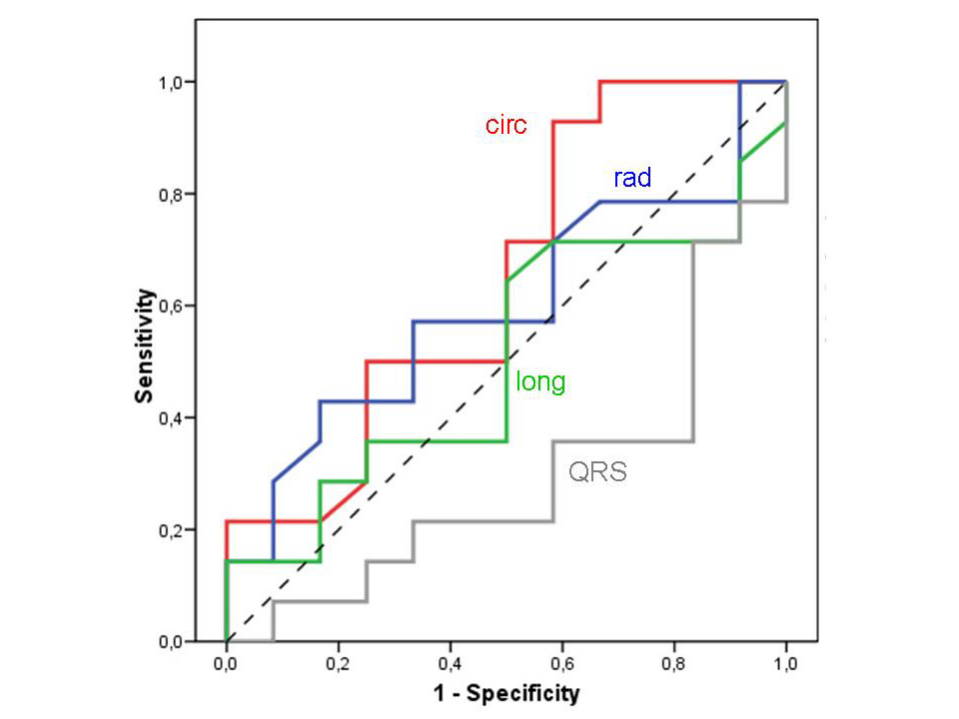
ROC curve analysis for the prediction of response to CRT (circ = circumferential 2D strain, max delay; long = longitudinal 2D strain, max delay, rad = radial 2D strain, max delay, QRS = QRS width at baseline).

ROC (Receiver Operator Characteristic) curve analysis (Figure 3) was performed to calculate the best cut-off value to predict response to CRT (Table 3). The area under the curve is 0.664 (± 0.144) for baseline maximum delay of circumferential 2D strain in the prediction of CRT response, which indicates a fair accuracy. The predictive value of circumferential 2D strain is not significant (p = 0.365) The best cut-off for this discrimination is a delay of 141.5 ms (sensitivity 93.3%, specificity 46.3%).

### Intra- and Interobserver variability

Intra- and interobserver reliabilities were calculated by the intraclass correlation coefficient (ICC). There were good correlations for Tissue Doppler measurements (ICC = 0.94, p < 0.001 and 0.96, p < 0.001, respectively) and circumferential (non-Doppler) 2 D strain measurements (ICC = 0.92, p < 0001 and 0.91, p < 0.001, respectively).

## Discussion

The main finding of this study is that circumferential 2D Strain delays decrease significantly in the responders but not in the non-responders to CRT. The analysis of circumferential 2D strain broadens the view of three-dimensional myocardial motion and indicates that circumferential myocardial synchrony is improved by CRT.

The majority of myocardial fibres have longitudinal orientation. Early in the development of heart failure, longitudinal function is impaired, with a compensation of radial and circumferential (= short axis) function [24]. The progressive dilated heart in heart failure is the result of impaired radial and circumferential function [25]. Our study indicates that circumferential function can be partially restored by CRT.

Circumferential asynchrony might play an important role in the measurement of overall asynchrony. A TAGGED-MRT-study found that asynchrony assessed by longitudinal motion is less sensitive, follows different time courses than circumferential asynchrony, and may manifest CRT benefit during specific cardiac phases depending on pacing mode. These results underscore the limitations of longitudinal analyses of asynchrony [26].

There are technical issues of circumferential 2D Strain of importance. The rather low frame rate (50–100/min) can impair the detection of myocardial contraction peaks. Despite the low temporal resolution of 2D-Strain, significant changes in our study and of previous studies [11,27,28] support that the method of 2D strain allows detection of myocardial asynchrony. The intra- and inter-observer variability is low and therefore, it seems unlikely that the observed differences in echocardiography are related to this.

This is supported by a recent study by Becker [27]. Circumferential 2D strain analysis was prospectively used to determine to optimal LV-lead position. The authors found that the optimal lead position led to more favourable reverse remodelling.

In contrast to our results, Zhang [28] found an improvement of the absolute value of circumferential (but not radial and longitudinal) 2D strain after three months of successful CRT. In our study, neither the longitudinal nor the radial or circumferential 2D strain values changed in the long-term follow up. We have used 2 D strain as a tool to measure asynchrony (i.e. we have analysed the maximum delays) and not of systolic myocardial performance (i.e. absolute values).

Neither circumferential nor radial and longitudinal 2D strain delays are able to predict CRT response. The 2 D Strain parameters are not superior to Tissue Doppler derived asynchrony. We conclude that no single parameter is able to help in patient selection for CRT.

The future role of echocardiography in selection of CRT candidates is not yet resolved. The recently published consensus statement of the American Society of Echocardiography points out that echocardiographic selection of CRT candidates should only be performed in borderline cases with difficult decision making [29].

The results from the CRT patients in our institution have led to the strategy currently used: an integration of seven parameters of inter- and intra-ventricular asynchrony in addition to the established parameters. If more than two parameters are indicative of myocardial asynchrony, a CRT indication supported by echocardiography is seen. We hypothesize that not single parameters of myocardial asynchrony but the integration of multiple echocardiographic parameters of asynchrony will facilitate the selection of CRT candidates.

In conclusion, circumferential 2D Strain delays decrease significantly after CRT, indicating an improvement in all three dimensions of myocardial contraction. However, the resulting predictive values of 2D strain delays are not superior to longitudinal and radial 2D-strain or TDI delays.

## Authors' contributions

FK and SS have equally contributed to the study. FK, SS, ACB, AG, SE participated in contributions to conception, analysis and interpretation of data, the follow-up echocardiographic examinations and made comments to the manuscript. HB has implanted the CRT systems and has analyzed data. BG has supervised and commented the study. ACB was the supervisor of echo examinations, is head of the echo lab, contributed by revising the manuscript critically.

## Supplementary Material

Additional file 1 Click here for file

Additional file 2 Click here for file
